# Association between serum Klotho levels and estimated pulse wave velocity in postmenopausal women: a cross-sectional study of NHANES 2007–2016

**DOI:** 10.3389/fendo.2024.1471548

**Published:** 2024-09-12

**Authors:** Baiqiang Wang, Wenqu Xu, Zeyuan Mei, Wei Yang, Xiao Meng, Guipeng An

**Affiliations:** ^1^ State Key Laboratory for Innovation and Transformation of Luobing Theory, Qilu Hospital of Shandong University, Jinan, China; ^2^ Key Laboratory of Cardiovascular Remodeling and Function Research, Chinese Ministry of Education, Chinese National Health Commission and Chinese Academy of Medical Sciences, Qilu Hospital of Shandong University, Jinan, China; ^3^ Department of Cardiology, Qilu Hospital of Shandong University, Jinan, China; ^4^ Department of Cardiology, People's Hospital of Rizhao, Rizhao, China

**Keywords:** serum Klotho, estimated pulse wave velocity, postmenopausal women, hypertension, arterial stiffness

## Abstract

**Background:**

Postmenopausal women are at an increased risk of arterial stiffness, which can be assessed using estimated pulse wave velocity (ePWV). This study aimed to investigate the relationship between serum klotho levels and ePWV in postmenopausal women.

**Methods:**

This cross-sectional study used data from postmenopausal women who participated in the National Health and Nutrition Examination Survey (NHANES) between 2007 and 2016. Participants were divided into two groups based on the presence of hypertension. Weighted multivariate linear regression was used to analyze the relationship between serum Klotho levels and ePWV in each group. Restricted cubic spline models with multivariable adjustments were employed to examine nonlinear associations within each group.

**Results:**

Our analysis included 4,468 postmenopausal women from the NHANES database, with 1,671 in the non-hypertensive group and 2,797 in the hypertensive group. In all regression models, serum Klotho (ln-transformed) levels were significantly and independently negatively correlated with ePWV in the non-hypertensive group. After fully adjusting for confounders, a 1-unit increase in ln(Klotho) was associated with a 0.13 m/s decrease in ePWV (β = −0.13, 95% CI −0.23 to −0.03; *p =* 0.008). Additionally, in the fully adjusted model, participants in the highest quartile of ln(Klotho) had an ePWV value 0.14 m/s lower than those in the lowest quartile (*p* for trend = 0.017; 95% CI −0.23 to −0.05; *p =* 0.002). This negative correlation was consistent across subgroups and was particularly significant among women aged < 60 years, nonsmokers, and non-Hispanic Black women. However, no association was observed between serum Klotho levels and ePWV in the hypertensive group.

**Conclusion:**

Hypertension may affect the relationship between serum Klotho level and ePWV in postmenopausal women. Increased serum Klotho levels may reduce arterial stiffness in postmenopausal women. Further studies are required to confirm these findings.

## Introduction

The risk of cardiovascular diseases (CVDs) increases with age in both men and women; however, in women, this risk accelerates more rapidly after menopause (1, 2). This transition often leads to conditions, such as central obesity, hypertension, diabetes, and dyslipidemia (3–5). Postmenopausal women experience substantial hormonal changes, particularly a reduction in estrogen levels, which are associated with an increased CVD risk. In an aging society, the burden of CVD among postmenopausal women is rising, leading to substantial medical and social costs (6). Thus, addressing the health status of postmenopausal women is of paramount importance.

Arterial stiffness increases with age and is recognized as an independent risk factor for cardiovascular morbidity and mortality (7). Accelerated arterial stiffness is a notable concern for postmenopausal women and is widely considered as an independent predictor of CVDs (8). Understanding the factors influencing arterial stiffness is crucial for identifying potential therapeutic targets to mitigate CVD risk in postmenopausal women. Measurement of arterial stiffness is recommended for the prevention and management of CVDs (9). Carotid-femoral pulse wave velocity (cf-PWV) is the standard method for assessing arterial stiffness; a higher cf-PWV indicates reduced vascular elasticity and increased arterial stiffness (10). However, cf-PWV is not been widely adopted in clinical practice due to the need of specialized personnel and equipment. To address these limitations, estimated pulse wave velocity (ePWV) has been introduced as an alternative method. ePWV, calculated using age and mean blood pressure (MBP), can effectively predict cf-PWV and has demonstrated an excellent correlation with *in vivo* assessments (11).

Serum Klotho is an anti-aging protein encoded by the Klotho gene (12). It is primarily expressed in the distal convoluted tubules of the kidneys and plays a critical role in various physiological processes, including inflammation regulation, antioxidation, and aging prevention (13, 14). Mice deficient in serum Klotho exhibit a range of syndromes similar to human aging, such as reduced lifespan, arterial stiffness, skin atrophy, and osteoporosis, whereas overexpression of serum Klotho extends the lifespan of transgenic mice by 30% (12, 15). Several cohort studies have indicated that decreased serum Klotho levels are associated with conditions, such as heart failure, hypertension, and atrial fibrillation (16–18). However, the relationship between serum Klotho levels and arterial stiffness in postmenopausal women remains unclear.

Therefore, our study aims to explore the relationship between serum Klotho levels and arterial stiffness in postmenopausal women, as assessed using ePWV.

## Methods

### Study population

**Figure 1:**
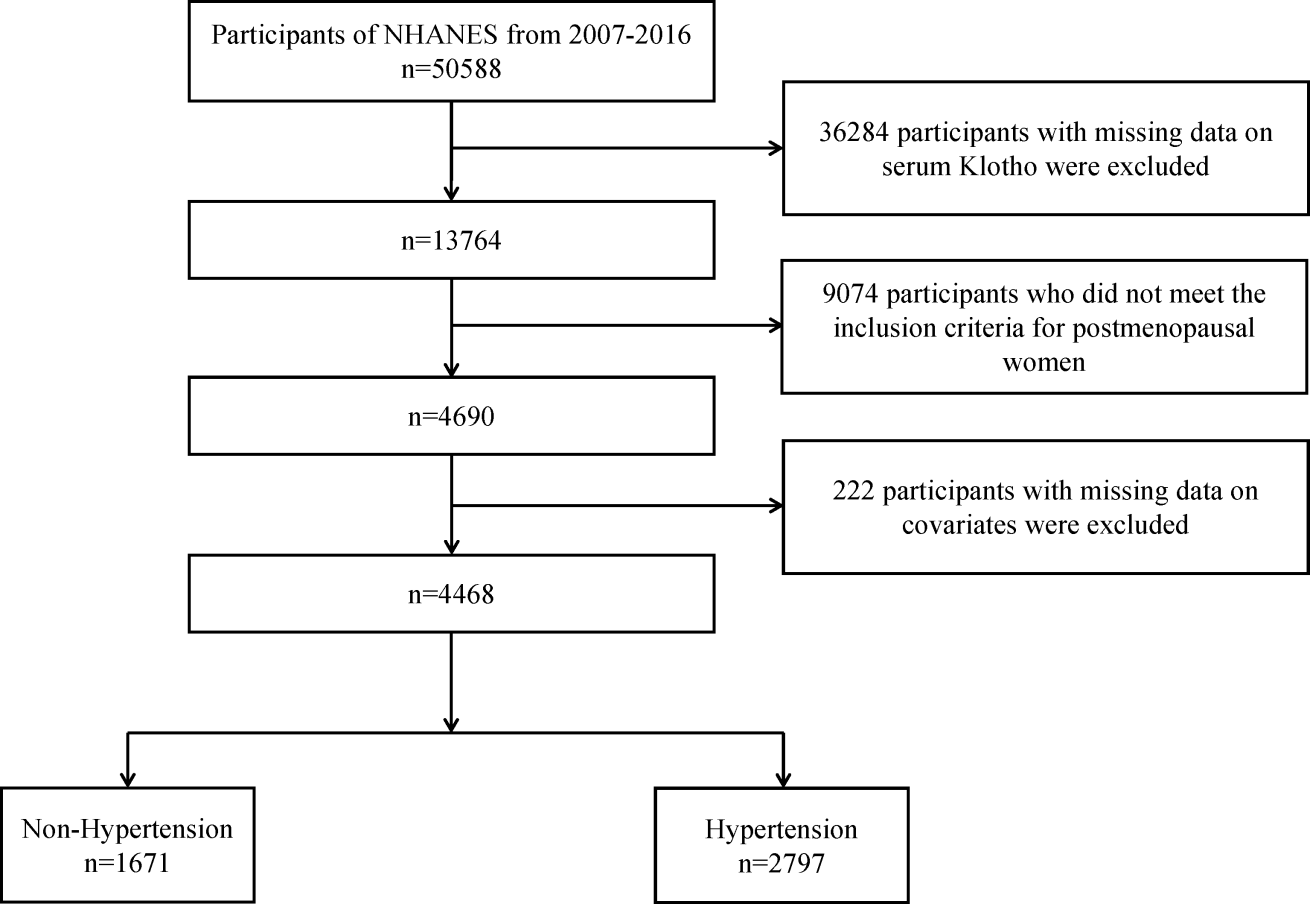
The flowchart of patients’ inclusion and exclusion.

This cross-sectional study, utilized data from the National Health and Nutrition Examination Survey (NHANES), an ongoing nationwide survey conducted by the National Center for Health Statistics (NCSH) at the U.S. Centers for Disease Control and Prevention. The NHANES adopts a multistage, stratified, subgroup probability sampling design in two-year cycles. Prior to conducting the present study, all participants provided written informed consent, and the study was approved by the National Centre for Health Statistics Ethics Review Board. For our study, we used data from five cycles involving 50,588 participants from 2007 to 2016. First, we excluded 36,284 participants with missing serum Klotho data, resulting in a cohort of 4,690 postmenopausal women. Next, we excluded 222 participants who lacked data on covariates, including heart failure, coronary heart disease, stroke, alcohol consumption, diabetes, smoking, hypertension, body mass index (BMI), estimated glomerular filtration rate (eGFR) and educational level. Ultimately, 4,468 postmenopausal women were included in the final analysis. Given the impact of hypertension and antihypertensive medications on arterial stiffness (19, 20), participants were further divided into hypertension (n=2,797) and non-hypertension groups (n=1,671). The participant selection process is illustrated in **Figure 1**.

### Evaluation of menopausal status

Menopausal status was defined based on the self-reported reproductive health questionnaire. Participants were classified as postmenopausal if they denied having a menstrual period within the past twelve months and subsequently indicated hysterectomy or menopause as the reason for this absence.

### Measurement of serum Klotho levels

Clinical samples available were collected by specialist workers on dry ice and stored at −80 °C. Samples from the participants were tested by enzyme-linked immunosorbent assay according to the manufacturer’s protocol. Each sample was analyzed in duplicate, and the average of the two values was used for the final result. A detailed description of the Klotho detection method is available on the NHANES website.

### Assessment of arterial stiffness

Blood pressure was measured by a trained health technician. Participants obtained three consecutive sphygmomanometric readings taken after 5 min of sitting still. Systolic blood pressure (SBP) and diastolic blood pressure (DBP) were determined by calculating the average of all blood pressure readings. The outcome variable for arterial stiffness was evaluated using ePWV, which was calculated using the following equation (11): 9.587 − (0.402 × age) + (4.560 × 10^−3^ × age^2^) − (2.621 × 10^−5^ × age^2^ × MBP) + (3.176 × 10^−3^ × age × MBP) − (1.832 × 10^−2^ × MBP). MBP was calculated as DBP + (0.4 × SBP − DBP).

### Assessment of covariates

Potential confounding variables that might affect the relationship between serum Klotho levels and arterial stiffness were considered. These included age, race/ethnicity, education level, smoking status, alcohol consumption, BMI, stroke, coronary heart disease, diabetes, hyperlipidemia, albumin level, and eGFR.

Education level was categorized into three groups: college or higher, high school or equivalent, and lower than high school. Race and ethnicity were classified as Mexican American, non-Hispanic Black, non-Hispanic White, or other. Smoking status was categorized according to the NCHS classification: individuals who smoked fewer than 100 cigarettes in their lifetime were considered never-smokers, whereas participants who smoked more than 100 cigarettes in their lifetime were considered smokers. For alcohol consumption, the categories were as follows: none (fewer than 12 drinks in a lifetime), former (more than 12 drinks in a lifetime but none in the past year), moderate (less than one drink per day for women), and heavy (one or more drinks per day for women). BMI was calculated as weight in kilograms (kg) divided by the square of the height in meters (m²). The eGFR was computed using the Chronic Kidney Disease Epidemiology Collaboration equation.

Hypertension was defined according to the 2017 American Heart Association blood pressure guidelines, encompassing individuals with a systolic blood pressure (SBP) ≥ 140 mmHg and/or diastolic blood pressure (DBP) ≥ 90 mmHg, those who self-reported hypertension, and those using antihypertensive medications (21). Coronary heart disease was identified as self-reported, doctor-diagnosed coronary heart disease, incidence of angina, or a history of heart attack. Hyperlipidemia was defined as total cholesterol levels ≥ 200 mg/dL, triglyceride levels ≥ 150 mg/dL, low-density lipoprotein levels ≥ 130 mg/dL, or high-density lipoprotein levels ≤ 50 mg/dL for women (22). Additionally, individuals who reported the use of cholesterol-lowering medications were categorized as having hyperlipidemia. Diabetes was defined as self-reported, doctor-diagnosed diabetes, or current use of insulin or antidiabetic medications.

### Statistical analysis

Given the complex multistage sampling design of the NHANES, appropriate sample weights (1/5 × 2 Year Mobile Examination Center Weight) were calculated according to the NHANES guidelines. Continuous variables are presented as weighted means ± standard error and compared using weighted independent sample t-tests. Categorical variables are presented as unweighted case numbers and weighted percentages and were compared using weighted chi-square tests. To address the skewed Klotho distributions and facilitate interpretation, the data were log transformed [ln(Klotho)]. Subsequently, ln(Klotho) was divided into four quartiles, with the first quartile serving as a reference. ln(Klotho) was analyzed as both a continuous and categorical variable using weighted multivariate linear regression models to examine the independent association between ln(Klotho) and ePWV in both the hypertensive and non-hypertensive groups. Model 1 did not adjust for covariates, while Model 2 was adjusted for age, race, educational level, and BMI. Model 3 included all the covariates from Model 2, along with adjustments for alcohol consumption, smoking status, diabetes mellitus, coronary heart disease, heart failure, stroke, hyperlipidemia, eGFR, and albumin levels. Multivariate-adjusted restricted cubic spline analysis was conducted to assess the nonlinear relationship between ln(Klotho) and ePWV in both groups. Additionally, stratified analyses were conducted based on age, BMI, hyperlipidemia, diabetes, race, and smoking status to examine the association between ln(Klotho) and ePWV across different subgroups. All analyses were conducted using R version 4.3.2, with *p* < 0.05 indicating statistical significance.

## Result

### Baseline characteristics

The study sample comprised 4,468 participants aged 40–79 years, recruited between 2007 and 2016. Among these 2,797 participants had hypertension, while 1,671 participants were normotensive. **Table 1** presents the baseline characteristics of the study population. Participants had a mean age of 61.0 ± 9.0 years. The results indicated, hypertensive patients were older than normotensive patients, with a higher prevalence of Black ethnicities, lower educational attainment, and lower family poverty income ratio. They also exhibited increased BMI and waist circumference. Moreover, the hypertensive group exhibited an increased incidence of comorbidities, such as diabetes, coronary heart disease, stroke, and heart failure. Hypertensive patients also exhibited elevated blood glucose and triglyceride levels, but reduced eGFR and total cholesterol levels (*p* < 0.001). Moreover, the hypertensive group showed increased ePWV (9.96 ± 1.64 vs. 8.72 ± 1.38, *p* < 0.001) and decreased serum Klotho levels (834 ± 288 vs. 866 ± 311, *p* = 0.021).

**Table 1:** Baseline characteristics of participants.

Characteristics	Overall	Non-Hypertension	Hypertension	*P* value
Number	4468	1671	2797	
Age (years)	61.0 ± 9.0	58.5 ± 8.6	63.1 ± 8.7	<0.001
Race (%)				<0.001
Non-Hispanic White	2019 (76.0)	831 (80.0)	1,188 (73.0)	
Non-Hispanic Black	876 (9.1)	209 (5.4)	667 (12.0)	
Mexican American	677 (5.2)	256 (5.1)	421 (5.2)	
Other Races	896 (9.3)	375 (9.1)	521 (9.5)	
Education level (%)				<0.001
College or above	2165 (60.0)	910 (65.0)	1255 (56.0)	
High school or equivalent	1059 (24.0)	375 (22.0)	684 (26.0)	
Less than high school	1244 (16.0)	386 (13.0)	858 (19.0)	
BMI (kg/m2)	30 ± 7	28 ± 6	31 ± 8	<0.001
Waist circumference (cm)	78 ± 19	73 ± 16	81 ± 20	<0.001
PIR	3.2 ± 1.6	3.5 ± 1.6	2.9 ± 1.6	<0.001
Heart failure (%)				<0.001
No	4301 (97.0)	1652 (99.0)	2649 (95.0)	
Yes	167 (3.2)	19 (0.9)	148 (5.0)	
CHD (%)				<0.001
No	4107 (93.0)	1609 (97.0)	2498 (91.0)	
Yes	361 (6.6)	62 (3.1)	299 (9.4)	
Hyperlipidemia (%)				0.500
No	1103 (24.0)	388 (23.0)	715 (25.0)	
Yes	3365 (76.0)	1283 (77.0)	2082 (75.0)	
DM (%)				<0.001
No	3553 (85.0)	1505 (93.0)	2048 (79.0)	
Yes	915 (15.0)	166 (6.6)	749 (21.0)	
Stroke (%)				<0.001
No	4223 (96.0)	1633 (98.0)	2590 (94.0)	
Yes	245 (4.3)	38 (2.0)	207 (6.0)	
Smoking (%)				0.200
No	2595 (55.0)	979 (57.0)	1616 (54.0)	
Yes	1873 (45.0)	692 (43.0)	1181 (46.0)	
Alcohol drinking (%)				<0.001
None	969 (15.0)	325 (14.0)	644 (17.0)	
Former	997 (19.0)	314 (15.0)	683 (22.0)	
Moderate	1379 (38.0)	554 (41.0)	825 (36.0)	
Heavy	1090 (28.0)	445 (30.0)	645 (26.0)	
Albumin (mg/dL)	4.23 ± 0.30	4.27 ± 0.29	4.20 ± 0.31	<0.001
Cholesterol (mg/dL)	210 ± 42	215 ± 39	206 ± 43	<0.001
Glucose (mg/dL)	104 ± 37	98 ± 27	108 ± 42	<0.001
Triglyceride (mg/dL)	156 ± 96	143 ± 84	167 ± 103	<0.001
eGFR (ml/min/1.73m2)	83 ± 22	86 ± 20	81 ± 23	<0.001
ePWV (m/s)	9.42 ± 1.65	8.72 ± 1.38	9.96 ± 1.64	<0.001
Klotho (pg/mL)	848 ± 299	866 ± 311	834 ± 288	0.021

All values were presented as mean ± SE, or counts (weighted, proportion).

BMI, body mass index; PIR, family income to poverty ratio; CHD, coronary heart disease; DM, diabetes mellitus; eGFR, glomerular filtration rate; ePWV, estimated pulse wave velocity.

### Associations between serum Klotho and ePWV in postmenopausal women with or without hypertension

**Figure 2:**
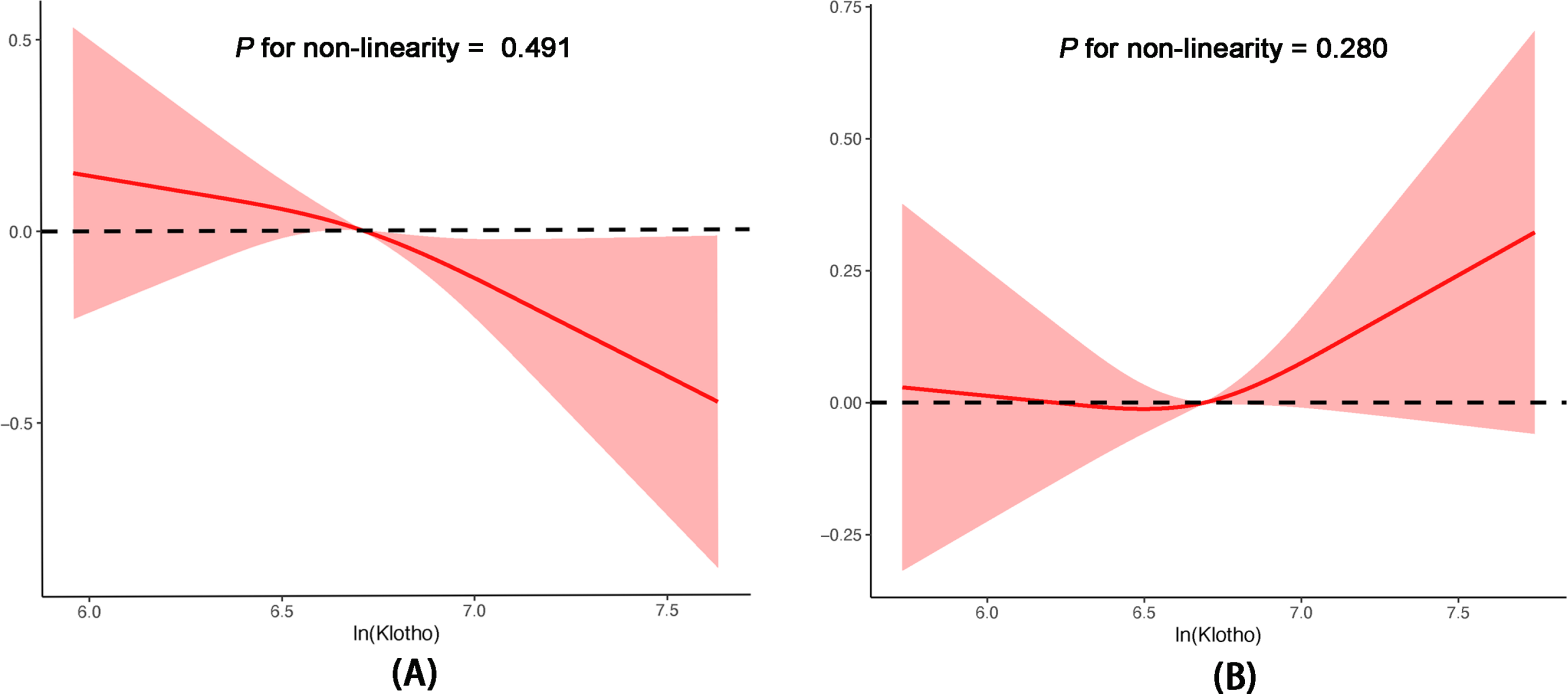
Associations between serum Klotho levels and ePWV in postmenopausal women without hypertension **(A)** and with hypertension **(B)** according to multivariable linear regression based on restricted cubic splines. The results were adjusted for age, race, education level, BMI, alcohol drinking, smoking status, DM, CHD, heart failure, stroke, hyperlipidemia, eGFR, and albumin. BMI, body mass index; CHD, coronary heart disease; DM, diabetes mellitus; eGFR, glomerular filtration rate; ePWV, estimated pulse wave velocity.

**Table 2** presents the β coefficients and corresponding 95% confidence intervals (CIs) for ln(Klotho) and ePWV across various models in the hypertensive and non-hypertensive groups. In the non-hypertensive group, ln(Klotho) and ePWV exhibited significant and independent negative correlations across the various adjusted models. After fully adjusting for confounders, a 1-unit increase in ln(Klotho) was associated with a decrease in ePWV by 0.13 m/s (β = −0.13, 95% CI −0.23 to −0.03; *p =* 0.008). Additionally, in the fully adjusted model, participants in the highest quartile of ln(Klotho) had an ePWV value 0.14 m/s lower than those in the lowest quartile of ln(Klotho) (*p* for trend = 0.017; 95% CI: −0.23 to −0.05; *p* = 0.002). However, this relationship was not observed in the hypertensive group. Restricted cubic spline models were used to further investigate the potential nonlinear relationship between ln(Klotho) and ePWV in both groups (**Figure 2**). The results suggested no nonlinear correlation in the hypertensive group (*p* for nonlinearity = 0.280).

**Table 2:** Associations between ln(Klotho) and ePWV in postmenopausal women with and without hypertension.

Variable	Model 1		Model 2		Model 3	
	β (95% CI)	*P* value	β (95% CI)	P value	β (95% CI)	*P* value
Non-Hypertension						
ln(Klotho)	-0.54 (-0.74, -0.33)	**<0.001**	-0.12 (-0.22, -0.02)	**0.017**	-0.13 (-0.23, -0.03)	**0.008**
ln(Klotho) quartiles						
Q1	Ref.		Ref.		Ref.	
Q2	-0.07 (-0.26, 0.11)	0.400	-0.08 (-0.17, 0.00)	0.057	-0.11 (-0.19, -0.02)	**0.011**
Q3	-0.10 (-0.29, 0.09)	0.300	-0.03 (-0.12, -0.06)	0.500	-0.04 (-0.13, 0.50)	0.300
Q4	-0.41 (-0.6,- 0.22)	**<0.001**	-0.12 (-0.21, -0.03)	**0.007**	-0.14 (-0.23, -0.05)	**0.002**
*P* for trend		**<0.001**		**0.031**		**0.017**
Hypertension						
ln(Klotho)	-0.19 (-0.37, 0.00)	**0.047**	0.05 (-0.06, 0.16)	0.300	0.03 (-0.08, 0.14)	0.600
ln(Klotho) quartiles						
Q1	Ref.		Ref.		Ref.	
Q2	-0.08 (-0.25, 0.09)	0.300	0.03 (-0.07, 0.13)	0.600	0.04 (-0.06, 0.14)	0.400
Q3	-0.50 (-0.22, 0.12)	0.500	-0.02 (-0.12, 0.08)	0.700	-0.03 (-0.13, 0.07)	0.500
Q4	-0.21 (-0.38, -0.04)	**0.017**	0.07 (-0.04, 0.17)	0.200	0.05 (-0.05, 0.16)	0.300
*P* for trend		**0.034**		0.400		0.600

Model 1: adjusted for none.

Model 2: adjusted for age, race, education level.

Model 3: adjusted for age, race, education level, BMI, alcohol drinking, smoking status, DM, CHD, heart failure, stroke, hyperlipidemia, eGFR, and albumin.

BMI, body mass index; CHD, coronary heart disease; DM, diabetes mellitus; eGFR, glomerular filtration rate; ePWV, estimated pulse wave velocity.

Bold values indicate statistical significance (P < 0.05).

### Subgroup analysis

**Figure 3:**
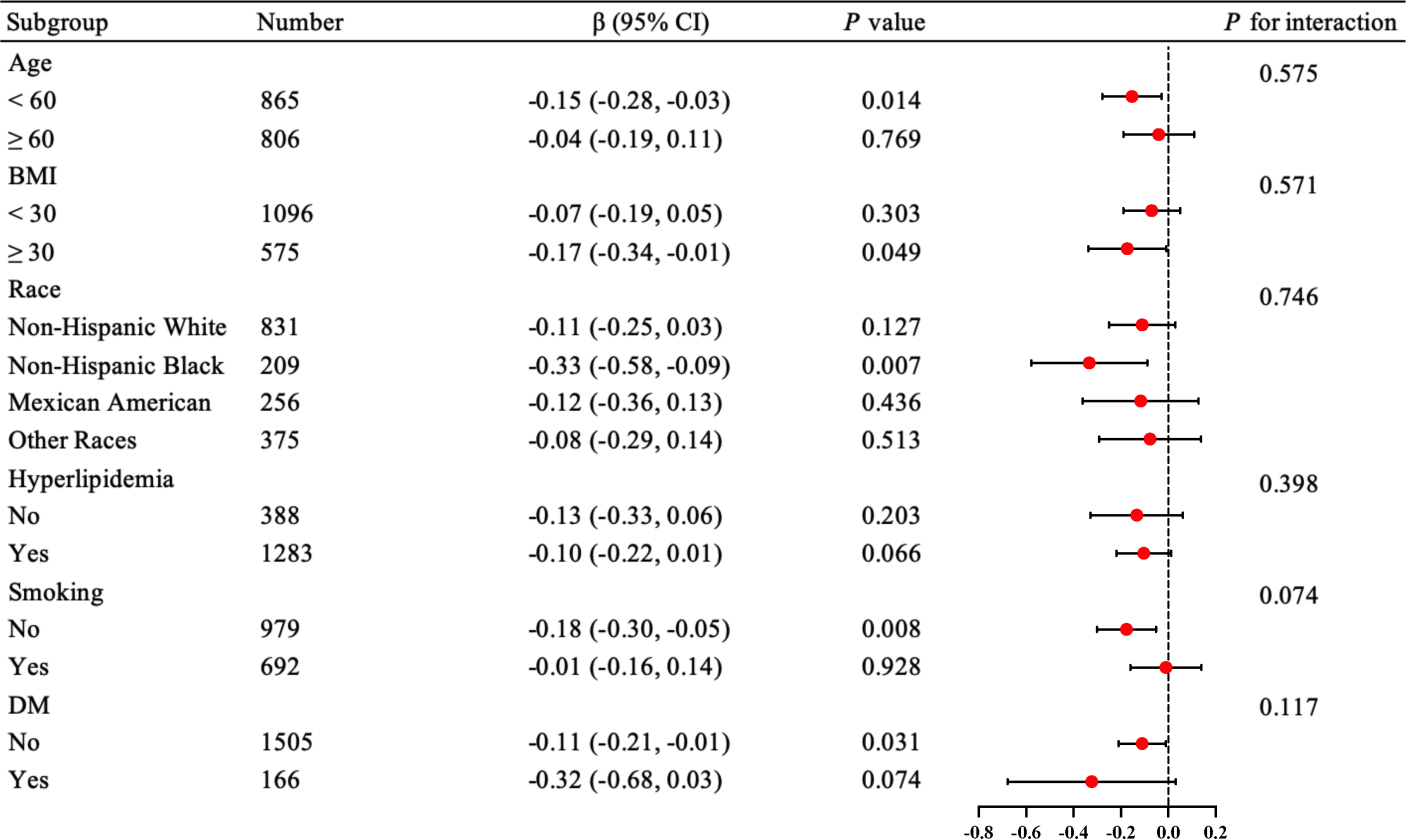
Forest plot for subgroup analysis of the relationship between serum Klotho levels and ePWV in postmenopausal women without hypertension. The results were adjusted for age, race, education level, BMI, alcohol drinking, smoking status, DM, CHD, heart failure, stroke, hyperlipidemia, eGFR, and albumin. BMI, body mass index (kg/m^2^); CHD, coronary heart disease; DM, diabetes mellitus; eGFR, glomerular filtration rate; ePWV, estimated pulse wave velocity.

We performed interaction and subgroup analyses of ln(Klotho) and ePWV in postmenopausal women without hypertension. In the fully adjusted model (Model 3), there was no significant interaction in the subgroup analysis stratified by age, BMI, race, smoking, hyperlipidemia, and diabetes (**Figure 3**). However, significant statistical differences were observed in patients younger than 60 years, non-smokers, and non-Hispanic Black patients (*p* = 0.014, *p* = 0.008, *p* = 0.007, respectively).

## Discussion

To the best of our knowledge, this is the first cross-sectional study exploring the relationship between serum Klotho levels and arterial stiffness in postmenopausal women. After adjusting for confounding factors, ln(Klotho) was found to be significantly negatively correlated with arterial stiffness in postmenopausal women without hypertension. This correlation remained consistent across various subgroups and showed statistically significant differences among women under 60 years of age, non-smokers, and non-Hispanic Black women. These findings suggest that early intervention to increase serum klotho levels and enhance risk factor management may be beneficial in preventing the progression of arterial stiffness in postmenopausal women.

Arterial stiffness is an indicator of vascular aging and is associated with various cardiovascular risk factors and comorbidities (7, 23). The European gold standard for assessing arterial stiffness is cf-PWV (24). However, cf-PWV measurement requires skilled operators and specialized equipment, which limits its application in clinical practice. Currently, ePWV is widely used as a novel index of arterial stiffness, and its accuracy in assessing arterial stiffness has been validated (11, 25). Postmenopausal women are at increased risk of arterial stiffness (26). It is widely recognized that a decline in blood estrogen levels is a key factor that exacerbates arterial stiffness in postmenopausal women (27). Estrogen deficiency reduces the repair capacity of endothelial cells and leads to arterial damage and endothelial dysfunction (28, 29). Although hormone replacement therapy (HRT) is commonly used in postmenopausal women, notable improvements in arterial stiffness have not been observed with standard HRT (30). Recent studies have suggested that age-related factors may play a crucial role in accelerating arterial stiffening in postmenopausal women.

In recent years, serum Klotho has garnered widespread attention as an anti-aging biomarker associated with longevity and various CVDs. Several studies have explored the relationship between serum Klotho levels and arterial stiffness. Animal model studies have demonstrated a causal relationship between serum Klotho deficiency and arterial stiffness measured using PWV (31). In a study of 114 patients with chronic kidney disease (CKD) patients, Kitagawa et al. found that low serum Klotho levels were independently associated with increased brachial-ankle pulse wave velocity (ba-PWV), suggesting that serum Klotho levels are a notable determinant of arterial stiffness in patients with CKD (32). Another cross-sectional cohort study that included 172 patients with early diabetic kidney disease, showed a statistically significant negative correlation between serum Klotho levels and pulse wave velocity (PWV), emphasizing the effect of serum Klotho levels on aortic wall stiffness (33). Analysis of the NHANES cohort from to 2007–2016 revealed an inverse and independent association between serum Klotho concentration and arterial stiffness, as indicated by pulse pressure (34). However, although this cohort was large, the pulse pressure is influenced by both cardiac and arterial functions. A more precise and reliable method for assessing arterial stiffness is PWV, which depends solely on arterial properties (35). Moreover, no relevant subgroup analyses targeting race or comorbidities have been performed.

Recently, conflicting results have been reported regarding the relationship between serum Klotho levels and arterial stiffness. In 2018, the KoreaN Cohort Study for Outcome in Patients With Chronic Kidney Disease (KNOW-CKD) trial analyzed data from 2,101 patients and found no association between serum Klotho levels and ba-PWV in patients with advanced CKD (36). Fountoulakis et al. suggested that this finding could be due to the influence of advanced CKD, diabetes, and the use of renin-angiotensin system inhibitors on serum Klotho levels and arterial stiffness (33). Additionally, Liang et al. conducted a study on 716 Chinese individuals and found no association between serum Klotho levels and cf-PWV (37). The exact cause of these discrepancies remains unclear, but they might be attributed to differences in ethnic populations, patient selection, and study design. Future research should include more diverse populations to further validate these findings.

Increased arterial stiffness has also been observed in postmenopausal women. In a small sample study, Matsubara et al. indicated a negative correlation between plasma Klotho levels and the β-stiffness index in postmenopausal women, suggesting that aerobic exercise training increased plasma Klotho levels and reduced arterial stiffness (38). In 2023, Yu et al. also found a significant negative correlation between serum Klotho concentration and hypertension in postmenopausal women (17). However, these studies had small sample sizes.

In our study, we analyzed a nationally representative sample of 4,468 postmenopausal women, which constitutes a large sample size. Additionally, this is the first study to use ePWV to assess arterial stiffness in postmenopausal women. We found a significant negative correlation between ln(Klotho) and ePWV in postmenopausal women without hypertension, independent of other cardiovascular risk factors. Different from previous studies, we found no correlation between serum Klotho levels and arterial stiffness in postmenopausal women with hypertension. Possible explanations include the following: First, previous studies with smaller sample sizes might have overlooked the impact of hypertension on the relationship between Klotho and ePWV. Second, hypertension interacts with arterial stiffness in a vicious cycle, potentially accelerating its progression (20, 39). Lastly, most participants with hypertension in our cohort received antihypertensive medications, which might have affected arterial stiffness (19).

Serum Klotho levels may be associated with arterial stiffness through several mechanisms. Inflammation and oxidative stress are potential causes of endothelial damage and arterial stiffness. Klotho can modulate inflammation by inhibiting TNF-α-induced expression of adhesion molecules (40) and NF-κB (41) activation. Additionally, decreased serum Klotho levels was significantly associated with a pro-inflammatory state, characterized by reduced serum IL-10 levels and elevated CRP levels and TNF-α/IL-10 ratio (42). In an *in vitro* experiment, Klotho deficiency increased the production of endogenous reactive oxygen species, promoting oxidative stress injury and apoptosis in mouse kidney cells (43). Recently, Donate-Correa et al. demonstrated that Klotho exerts its antioxidant effects through various pathways, including the regulation of manganese superoxide dismutase, the transcription factors FoxO and Nrf2, and other known antioxidant systems (44). Therefore, Klotho is a potential therapeutic target for oxidative stress. Furthermore, Klotho inhibited vascular calcification by preventing the transformation of muscle cells into osteoblast-like cells (45). Moreover, studies have demonstrated the protective effects of serum Klotho against angiotensin II-mediated oxidative stress, apoptosis, and senescence in human aortic smooth muscle cells (46). Mechanistic studies demonstrated that Klotho knockdown potentiated the development of accelerated calcification through a Runx2 and myocardin-serum response factor-dependent pathway (47). Other mechanisms by which Klotho deficiency leads to arterial stiffness include enhanced autophagic activity, which results in the upregulation of scleraxis, a key transcription factor for collagen synthesis (48). In animal studies, Klotho-deficient mice exhibited impaired gonadotropin regulation, leading to atrophy of the female reproductive system and reduced estrogen synthesis (49). This suggests that Klotho affects arterial stiffness by modulating estrogen levels, which may explain the association between serum Klotho and arterial stiffness in postmenopausal women. Further studies are required to explore these underlying mechanisms.

Finally, we assessed the stability of our results using subgroup and interaction tests; however, no interactions were observed. We found a significant negative correlation between ln(Klotho) and ePWV among women aged < 60 years, non-smokers, and non-Hispanic Black women. Age is a significant factor influencing arterial stiffness and calcification (50). Age-related extracellular matrix stiffening can trigger pathogenic mechanotransductive signaling, leading to Klotho promoter methylation, which in turn downregulates Klotho gene expression and accelerates chondrocyte aging *in vitro* (51). These mechanisms may explain the impact of age on the predictive value of serum Klotho. These findings suggest that early interventions aimed at increasing serum Klotho levels may be beneficial in preventing the progression of arterial stiffness and hypertension in postmenopausal women.

In recent years, therapies targeting Klotho have shown great potential. Animal studies have demonstrated that enhancing Klotho expression can inhibit the progression of hypertension and mitigate kidney damage (52). Repeated low-dose injections of Klotho (10 μg/kg) significantly inhibited the growth of breast tumors in mice, and Klotho was well tolerated (53). In 2023, Castner et al. discovered for the first time in non-human primates that a single low-dose injection of Klotho (10 μg/kg) significantly improved cognitive function in aging rhesus monkeys (54). Further animal experiments and studies are required to demonstrate the effect of serum Klotho on improving arterial stiffness in postmenopausal women.

The present study explored the complex relationship between serum Klotho levels and ePWV in a nationally representative sample of postmenopausal women. However, our study has several limitations. First, its cross-sectional design precluded the establishment of a causal relationship between serum Klotho levels and arterial stiffness. Second, some data were collected through self-reported measures, which may have affected the accuracy. Finally, despite our efforts to account for various potential confounding factors, the possibility of residual confounding remains, which could have affected the validity of our results.

## Conclusion

In non-hypertensive postmenopausal women, serum Klotho levels were significantly negatively correlated with ePWV, particularly among women aged < 60 years, nonsmokers, and non-Hispanic Black women. Future research is necessary to further explore the causal mechanisms with the aim of improving arterial stiffness and decreasing cardiovascular risk in postmenopausal women.

## Data Availability

Publicly available datasets were analyzed in this study. This data can be found here: https://www.cdc.gov/nchs/nhanes/.
